# Safety and Immunogenicity of Respiratory Syncytial Virus Prefusion F Protein Vaccine when Co-administered with Adjuvanted Seasonal Quadrivalent Influenza Vaccine in Older Adults: A Phase 3 Randomized Trial

**DOI:** 10.1093/cid/ciae365

**Published:** 2024-08-05

**Authors:** Rebecca Clark, Sam Davies, Jorge Labrador, Paul Loubet, Silvina Natalini Martínez, Helena Moza Moríñigo, Jean-François Nicolas, Mercè Pérez Vera, Mika Rämet, Maria Henar Rebollo-Rodrigo, Iván Sanz-Muñoz, Nancy Dezutter, Sophie Germain, Marie-Pierre David, Amulya Jayadev, Hiwot Amare Hailemariam, Shady Kotb, Nadia Meyer

**Affiliations:** Layton Medical Centre, Blackpool, United Kingdom; South Gloucestershire Medical Research Unit, Bristol, United Kingdom; Research Unit, Hospital Universitario de Burgos, Burgos, Spain; Department of Infectious and Tropical Diseases, VBIC (Bacterial Virulence and Chronic Infection), INSERM, University of Montpellier, CHU Nimes, Nimes, France; Unidad de Investigación de Vacunas, Instituto de Investigación Sanitaria HM, Madrid, Spain; Departamento de Medicina Preventiva y Salud Pública, Hospital Universitario Fundación Jiménez Díaz, Madrid, Spain; Department of Allergology of Clinical Immunology, Lyon Sud University Hospital, CIRI, INSERM U1111, University Lyon1, Pierre-Bénite, France; ABS La Roca del Vallès, Barcelona, Spain; Finnish Vaccine Research Ltd. and Faculty of Medicine and Health Technology, Tampere University, Tampere, Finland; Servicio de Medicina Preventiva y Salud Pública, University Hospital Marqués de Valdecilla, Santander, Cantabria, Spain; National Influenza Centre, Valladolid, Spain; Instituto de Estudios de Ciencias de la Salud de Castilla y León, Soria, Spain; GSK, Wavre, Belgium; GSK, Wavre, Belgium; GSK, Wavre, Belgium; GSK, Bangalore, India; GSK, Wavre, Belgium; GSK, Wavre, Belgium; GSK, Wavre, Belgium

**Keywords:** RSVPreF3 OA, adjuvanted seasonal influenza vaccine, concurrent administration, immunogenicity, safety

## Abstract

**Background:**

We evaluated co-administration of adjuvanted seasonal quadrivalent influenza vaccine (FLU-aQIV) and respiratory syncytial virus (RSV) prefusion F protein-based vaccine (RSVPreF3 OA) in ≥65-year-olds.

**Methods:**

This phase 3, open-label trial randomized ≥65-year-olds to receive FLU-aQIV and RSVPreF3 OA concomitantly (Co-Ad) or sequentially, 1 month apart (Control). Primary objectives were to demonstrate the non-inferiority of FLU-aQIV and RSVPreF3 OA co-administration versus sequential administration in terms of hemagglutination inhibition (HI) titers for each FLU-aQIV strain and RSV-A and RSV-B neutralization titers, 1 month post-vaccination. Reactogenicity and safety were also assessed.

**Results:**

Overall, 1045 participants were vaccinated (Co-Ad: 523; Control: 522). Non-inferiority of FLU-aQIV and RSVPreF3 OA co-administration versus sequential administration was demonstrated in terms of HI titers for the A/Victoria(H1N1), B/Victoria, and B/Yamagata influenza strains and RSV-A neutralization titers (upper limits [ULs] of 95% confidence intervals [CIs] for adjusted geometric mean titer [GMT] ratios [Control/Co-Ad] ≤1.50) but not for A/Darwin(H3N2) HI titers (95% CI UL = 1.53). The immune response to A/Darwin(H3N2) was further assessed post-hoc using a microneutralization assay; the post-vaccination adjusted GMT ratio (Control/Co-Ad) was 1.23 (95% CI: 1.06–1.42, ie, UL ≤1.50), suggesting an adequate immune response to A/Darwin(H3N2) following co-administration. RSV-B neutralization titers were comparable between groups (95% CI UL for adjusted GMT ratio ≤1.50). Solicited adverse events were mostly mild or moderate and transient; unsolicited and serious adverse event rates were balanced between groups.

**Conclusions:**

Adjuvanted FLU-aQIV and RSVPreF3 OA had acceptable reactogenicity/safety profiles when co-administered in ≥65-year-olds, without clinically relevant interference with the immune responses to either vaccine.

**Clinical Trials Registration:**

NCT05568797

## Teaser

Respiratory syncytial virus prefusion F protein-based vaccine (RSVPreF3 OA) and adjuvanted seasonal quadrivalent influenza vaccine had acceptable safety profiles when co-administered in adults ≥65 years of age, without clinically relevant interference with the immune responses to either vaccine.

## Graphical Abstract

**Graphical Abstract:**
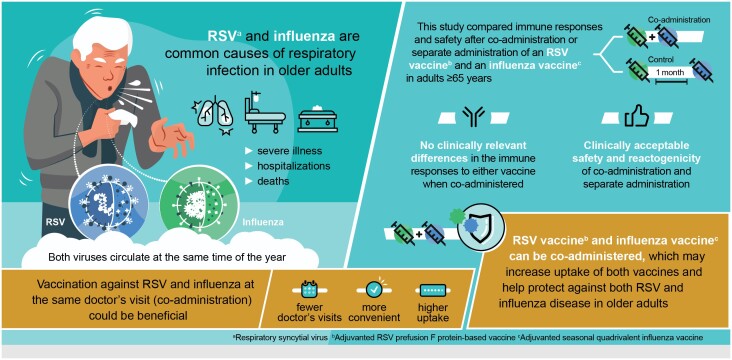

**(See the Major Article by Ferguson et al. on pages 1074–84; Editorial Commentary by Branche on pages 1099–101.)**

Respiratory syncytial virus (RSV) is a major cause of acute respiratory illness [1]. In addition to infants, people of advanced age and those with certain underlying medical conditions (eg, heart or lung disease and diabetes) are at increased risk for severe RSV disease [2–6]. The adjuvanted RSV prefusion F protein-based vaccine (RSVPreF3 OA, *Arexvy*, GSK) demonstrated efficacy against RSV-related lower respiratory tract disease, was immunogenic, and had an acceptable safety profile in adults ≥60 years old [7, 8].

Older adults and people with diabetes, cardiopulmonary and other chronic conditions are also at increased risk of severe outcomes and complications of influenza [9, 10]. Due to immunosenescence, influenza vaccines are often less effective in ≥65-year-olds than in younger people [11, 12]. High-dose and adjuvanted seasonal influenza vaccines have been shown to improve effectiveness compared to conventional vaccines [13–15] and are therefore recommended in older adults [10, 16].

Influenza and RSV viruses co-circulate, and in regions with temperate climates, seasonal epidemics of both viruses occur mainly during winter months [17, 18]. In these regions, seasonal influenza vaccination is therefore typically offered in early autumn [10, 16, 19], which is when RSV vaccination may also be recommended [6, 20, 21]. Co-administration of influenza and RSV vaccines during a single consultation could help protect older adults against both infections, while reducing the number of doctor's appointments, improving convenience, and thereby increasing vaccination coverage [22]. However, for co-administration of 2 vaccines to be recommended, it should not impact the immunogenicity and safety of either vaccine.

Co-administration of RSVPreF3 OA with non-adjuvanted (conventional and high-dose) seasonal quadrivalent influenza vaccines was recently evaluated [23, 24]. The current study assessed the immunogenicity, reactogenicity, and safety of co-administration versus sequential administration of RSVPreF3 OA and the adjuvanted inactivated seasonal quadrivalent influenza vaccine (FLU-aQIV, *Fluad Tetra/FLUAD QUADRIVALENT/Fluad* Quad, Seqirus) in adults aged ≥65 years.

## METHODS

### Study Design, Participants, and Interventions

This phase 3, randomized, open-label trial (ClinicalTrials.gov: NCT05568797) took place in 37 centers in Belgium, Finland, France, Spain, and the United Kingdom. The study was conducted in accordance with the Declaration of Helsinki, Good Clinical Practice, and regulatory requirements. The protocol, approved by the relevant ethics committees, is available on https://www.gsk-studyregister.com/en/trial-details/?id=218350.

Adults aged ≥65 years, including those with chronic stable medical conditions (with/without specific treatment), were enrolled. Individuals with confirmed/suspected immunosuppressive or immunodeficient conditions resulting from disease or therapy, individuals who had received an influenza vaccine within 6 months before FLU-aQIV administration in the study, and those who had previously received an RSV vaccine were excluded. All participants provided written/witnessed informed consent before study-specific procedure was performed. Eligibility criteria and enrollment rules are detailed in the Supplementary methods.

**Figure 1.**
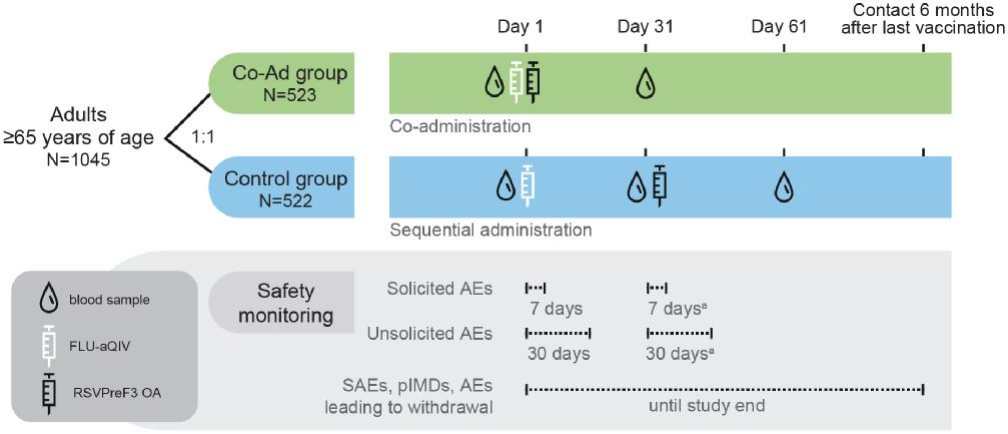
Study design. Abbreviations: AE, adverse event; FLU-aQIV, adjuvanted inactivated seasonal quadrivalent influenza vaccine; pIMD, potential immune-mediated disease; RSVPreF3 OA, respiratory syncytial virus prefusion F protein-based vaccine; SAE, serious adverse event. ^a^Applies to Control group only.

Participants were randomly assigned, 1:1, to receive RSVPreF3 OA concomitantly with FLU-aQIV on day 1 (Co-Ad group); or FLU-aQIV on day 1 and RSVPreF3 OA on day 31 (Control group) (Figure 1). Vaccines were injected in the deltoid muscle (of opposite arms when co-administered). An RSVPreF3 OA dose contained 120 µg RSVPreF3 antigen and AS01_E_ adjuvant. FLU-aQIV contained 15 µg hemagglutinin each of 2 influenza A-like strains (A/Victoria [H1N1] and A/Darwin [H3N2]) and 2 influenza B-like strains (from B/Victoria and B/Yamagata lineages), and MF59C.1 adjuvant. Strains were those recommended by the World Health Organization for the 2022/2023 Northern-Hemisphere influenza season [25] (Supplementary methods). Laboratories performing sample testing were blinded.

### Objectives

Primary objectives were to demonstrate the non-inferiority of the immune responses to FLU-aQIV and RSVPreF3 OA when co-administered versus when administered sequentially, in terms of hemagglutination inhibition (HI) titers for each FLU-aQIV vaccine strain and in terms of RSV-A and RSV-B neutralization titers, 1 month post-vaccination. Secondary objectives included assessment of non-inferiority of co-administration versus sequential administration in terms of seroconversion rates for each FLU-aQIV vaccine strain, evaluation of the humoral immune responses to both vaccines, reactogenicity, and safety.

### Immunogenicity Assessments

Blood samples from Co-Ad participants were collected on day 1 (pre-vaccination) and day 31 (1 month post-FLU-aQIV/RSVPreF3 OA co-administration). For Control participants, blood samples were collected on day 1 (pre-FLU-aQIV), day 31 (1 month post-FLU-aQIV/pre-RSVPreF3 OA), and day 61 (1 month post-RSVPreF3 OA) (Figure 1).

Humoral responses to FLU-aQIV were measured using HI assays for each influenza vaccine strain [26]. Humoral responses to RSVPreF3 OA were measured using neutralization assays for RSV-A and RSV-B [7]. To further characterize the immune response to A/Darwin (H3N2), a post-hoc analysis was performed using a microneutralization (MN) assay for this strain [27]. Assay cut-offs are included in the Supplementary methods.

### Reactogenicity and Safety Assessments

Participants recorded solicited adverse events (AEs) starting within 7 days after each dose in electronic diaries. Unsolicited AEs starting within 30 days after each dose and serious AEs (SAEs) and potential immune-mediated diseases (pIMDs) occurring from day 1 until study end (∼6 months post-last vaccination) were collected through questioning at study visits/contacts. The severity of AEs was graded on a scale from 1 (mild) to 3 (severe). Fever was defined as an oral or axillary temperature ≥38.0 °C. Solicited AEs were all considered as causally related to vaccination. For the other AEs, the investigators used their clinical judgment to assess causality.

### Statistical Analyses

Statistical analyses were performed using SAS Drug Development. Target enrollment was 514 participants per group (Supplementary methods).

Immunogenicity was assessed in the per-protocol set (all participants who received at least 1 [Control] or all [Co-Ad] study vaccinations per protocol, had immunogenicity results pre- and post-vaccination, complied with blood draw intervals, had no medical conditions that could interfere with immunogenicity, and did not receive prohibited medication/vaccination).

The confirmatory primary objectives were tested sequentially to control the overall type I error at 2.5% (1-sided). First, non-inferiority of FLU-aQIV and RSVPreF3 OA co-administration versus sequential administration in terms of influenza HI titers and RSV-A neutralization titers was tested and demonstrated if the upper limits (ULs) of the 2-sided 95% confidence intervals (CIs) for the adjusted geometric mean titer (GMT) ratios (Control/Co-Ad) for each FLU-aQIV strain and for RSV-A were ≤1.50 at 1 month post-vaccination. If this was met, the non-inferiority of co-administration versus sequential administration in terms of RSV-B neutralization titers was tested and demonstrated based on the same criterion (Supplementary methods).

All other objectives were evaluated descriptively without adjustments for multiplicity. Non-inferiority of co-administration versus sequential administration in terms of seroconversion rate for HI antibodies was assessed through the 2-sided 95% CI for the difference in seroconversion rates (SCRs) (Control minus Co-Ad) for each FLU-aQIV strain (reference criterion: UL of 95% CI ≤10%) at 1 month post-vaccination. HI GMTs and mean geometric increases (MGIs, ie, geometric means of the within-participant ratios of the post-vaccination over the pre-vaccination titers) for each FLU-aQIV strain, and RSV-A/RSV-B neutralization GMTs and MGIs were calculated with 95% CIs. SCRs (percentages of participants with an HI pre-vaccination titer <1:10 and post-vaccination titer ≥1:40 or pre-vaccination titer ≥1:10 and ≥4-fold increase in post-vaccination titer) and seroprotection rates (SPRs, percentages of participants with HI titer ≥1:40; often used as a surrogate marker of protection against influenza [10, 28]) were calculated with exact 95% CIs. SCRs and SPRs for the MN assay were defined using the same cut-offs.

The Center for Biologics Evaluation and Research (CBER) and Committee for Medicinal Products for Human Use (CHMP) criteria for seroprotection and seroconversion for HI antibodies were also assessed. To meet CBER criteria (for ≥65-year-olds), the lower limits (LLs) of the 95% CIs for the SCRs should be ≥30% and for SPRs ≥60% [29]. To meet CHMP criteria (for ≥60-year-olds), either SPRs should be >60%, or SCRs >30%, or MGIs >2.0 for each strain [30].

Safety and reactogenicity were analyzed in the exposed set (participants who received a study vaccination).

## RESULTS

### Study Participants

**Figure 2.**
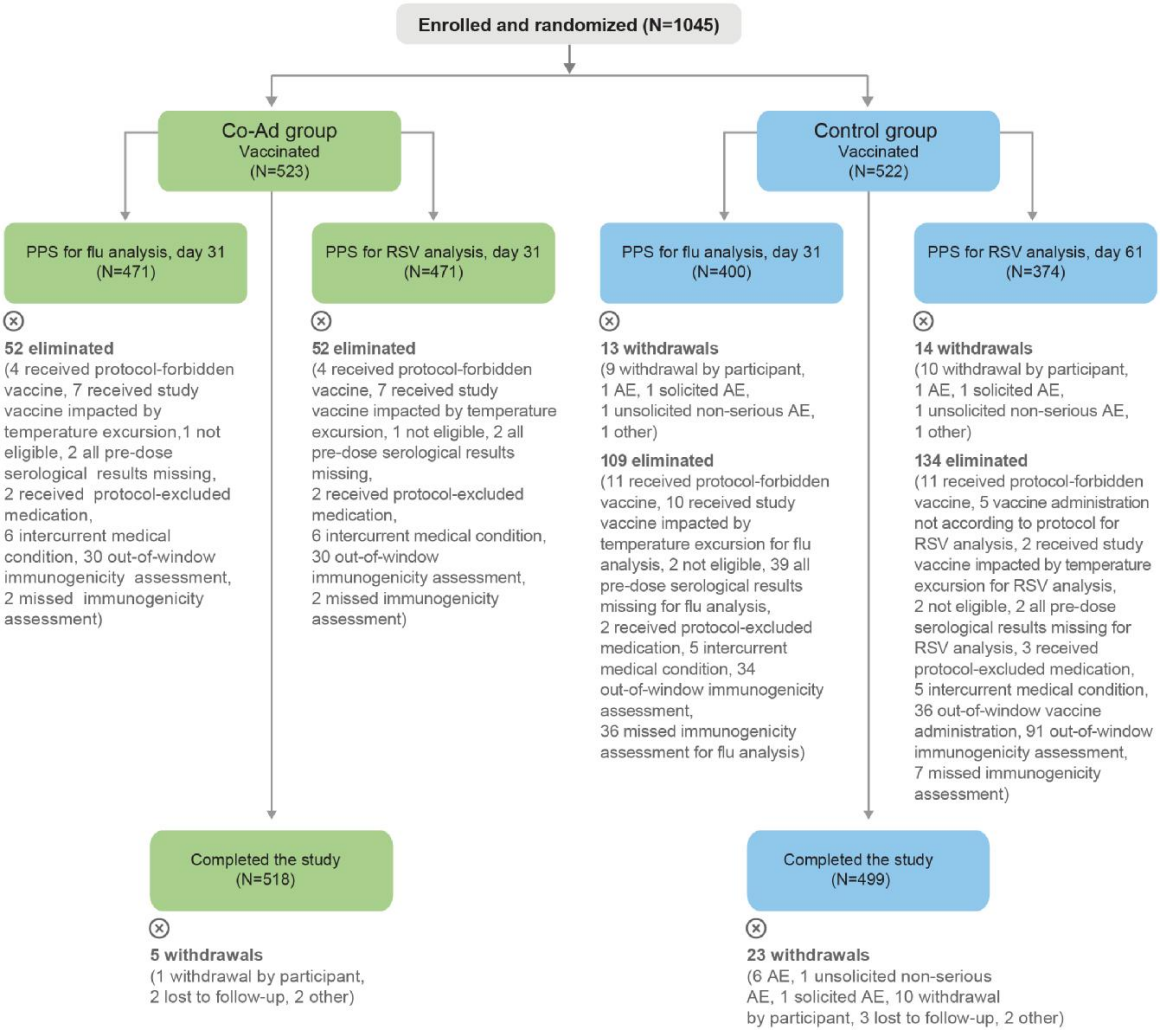
Flow of participants. For eliminations from the PPS, multiple reasons could apply for 1 participant; all reasons are listed in the figure. Co-Ad, group of participants who received RSVPreF3 OA and FLU-aQIV concomitantly on day 1. Control, group of participants who received FLU-aQIV on day 1 and RSVPreF3 OA on day 31; N, number of participants in the indicated analysis set and group. Abbreviations: AE, adverse event; FLU-aQIV, adjuvanted inactivated seasonal quadrivalent influenza vaccine; PPS, per-protocol sets for immunogenicity; RSVPreF3 OA, respiratory syncytial virus (RSV) prefusion F protein-based vaccine.

The study took place from 14 October 2022 to 17 July 2023. In total, 1045 participants were enrolled and vaccinated: 523 in the Co-Ad and 522 in the Control group; 1017/1045 participants (97.3%) completed the study (ie, safety contact ∼6 months post-last vaccination) (Figure 2). The per-protocol sets included 471 Co-Ad and 400 Control participants for the post-vaccination flu analysis and 471 Co-Ad and 374 Control participants for the post-vaccination RSV analysis. Baseline characteristics were balanced between the 2 groups in the exposed set and per-protocol sets (Table 1, Supplementary Table 1). The occurrence of pre-existing conditions associated with an increased risk for RSV and influenza disease was also balanced between groups (Supplementary Table 2).

**Table 1.** Baseline Characteristics of the Participants (Exposed Set)

Characteristic	Co-Ad N = 523	Control N = 522
Mean age (SD), y	72.1 (5.4)	72.2 (5.2)
Age group, n (%)		
65–69 y	200 (38.2)	198 (37.9)
70–79 y	269 (51.4)	271 (51.9)
≥80 y	54 (10.3)	53 (10.2)
Sex, n (%)		
Female	268 (51.2)	247 (47.3)
Male	255 (48.8)	275 (52.7)
Race, n (%)		
Asian	0 (0.0)	1 (0.2)
Black	0 (0.0)	1 (0.2)
White	522 (99.8)	516 (98.9)
Other	1 (0.2)	3 (0.6)
Unknown	0 (0.0)	1 (0.2)
Country, n (%)		
Belgium	60 (11.5)	61 (11.7)
Finland	55 (10.5)	55 (10.5)
France	111 (21.2)	112 (21.5)
Spain	224 (42.8)	220 (42.1)
United Kingdom	73 (14.0)	74 (14.2)
Any pre-existing condition^a^	488 (93.3)	496 (95.0)

Abbreviations: Co-Ad, group of participants who received RSVPreF3 OA and FLU-aQIV concomitantly on day 1; Control, group of participants who received FLU-aQIV on day 1 and RSVPreF3 OA on day 31; FLU-aQIV, adjuvanted inactivated seasonal quadrivalent influenza vaccine; N, number of participants in the exposed set; n (%), number (percentage) of participants in the indicated category. RSVPreF3 OA, respiratory syncytial virus (RSV) prefusion F protein-based vaccine; SD, standard deviation.

^a^Any pre-existing medical condition based on the participant's medical history obtained by interviewing the participant and/or reviewing the participant's medical records. The most common conditions were hypertension (Co-Ad: 45.3%, Control: 44.6%) and hypercholesterolemia (Co-Ad: 22.2%; Control: 21.5%). Additional data related to pre-existing medical conditions are included in Supplementary Table 2.

### Immunogenicity

**Figure 3.**
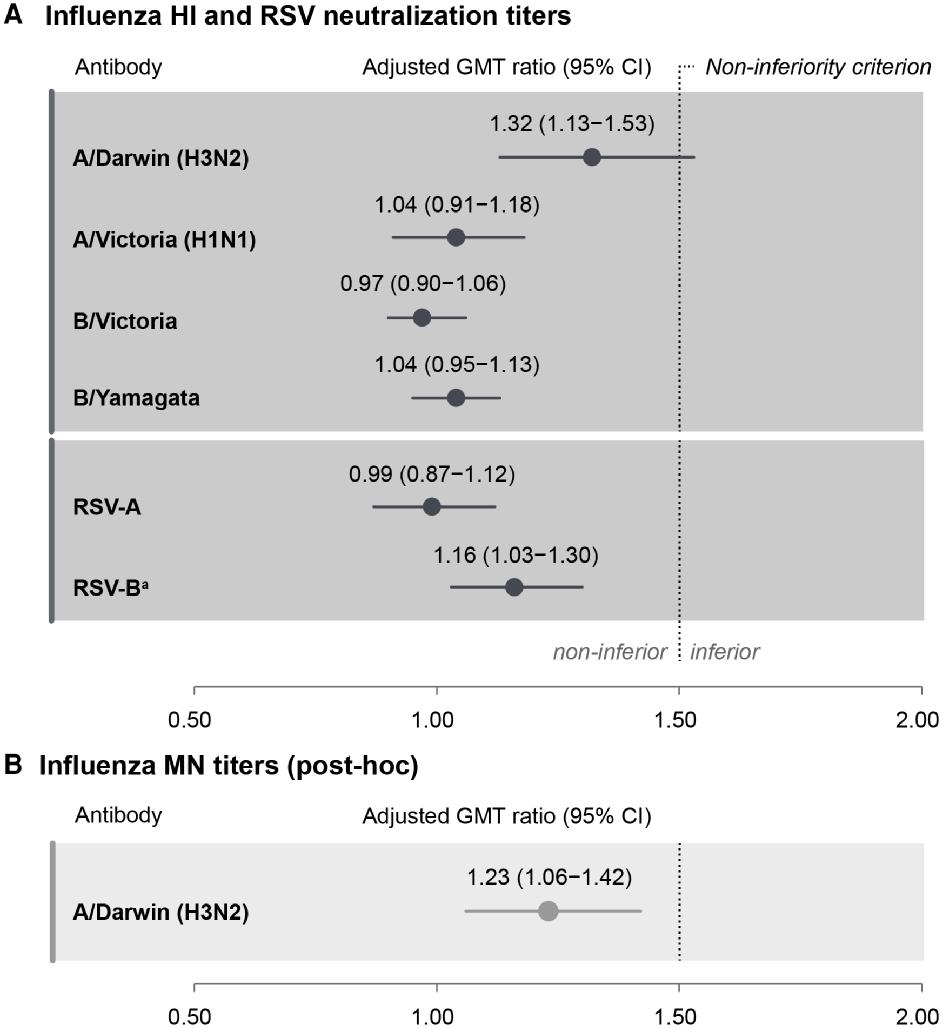
Non-inferiority of FLU-aQIV and RSVPreF3 OA co-administration versus sequential administration, evaluated by adjusted GMT ratios for influenza HI and RSV neutralizing titers *A*, as well as influenza MN titers for A/Darwin (H3N2) *B*, (per-protocol sets for flu and RSV analyses). CIs are depicted as error bars. Success criterion for non-inferiority: upper limits of 2-sided 95% CIs for adjusted geometric mean titer (GMT) ratios (Control/Co-Ad) for each FLU-aQIV strain and for RSV-A and RSV-B were ≤1.50 at 1 m post-vaccination. Abbreviations: CI, confidence interval; FLU-aQIV, adjuvanted inactivated seasonal quadrivalent influenza vaccine; HI, hemagglutination inhibition; MN, microneutralization; RSVPreF3 OA, respiratory syncytial virus (RSV) prefusion F protein-based vaccine. ^a^As the non-inferiority analysis was performed sequentially, and the success criterion was not met for A/Darwin (H3N2), non-inferiority for RSV-B neutralization titers was no longer a confirmatory endpoint and was evaluated descriptively.

Non-inferiority of FLU-aQIV and RSVPreF3 OA co-administration versus sequential administration was demonstrated in terms of HI titers for the A/Victoria (H1N1), B/Victoria, and B/Yamagata influenza strains and in terms of RSV-A neutralization titers 1 month post-vaccination (ULs of 95% CIs for adjusted GMT ratios were ≤1.50) but not in terms of HI titers for A/Darwin (H3N2) (UL of 95% CI was 1.53) (Figure 3*A*). Because the non-inferiority analysis was performed sequentially and the success criteria for the first step (demonstrating non-inferiority for all influenza strains and RSV-A) were not met for all influenza strains, the second step (demonstrating non-inferiority for RSV-B) was not considered a confirmatory endpoint and was evaluated descriptively instead. RSV-B neutralization GMTs 1 month post-RSVPreF3 OA vaccination were comparable between groups, and the UL of the 95% CI for the adjusted GMT ratio was ≤1.50 (Figure 3*A*). Results in the exposed set were similar to those in the per-protocol sets (Supplementary Table 3).

**Figure 4.**
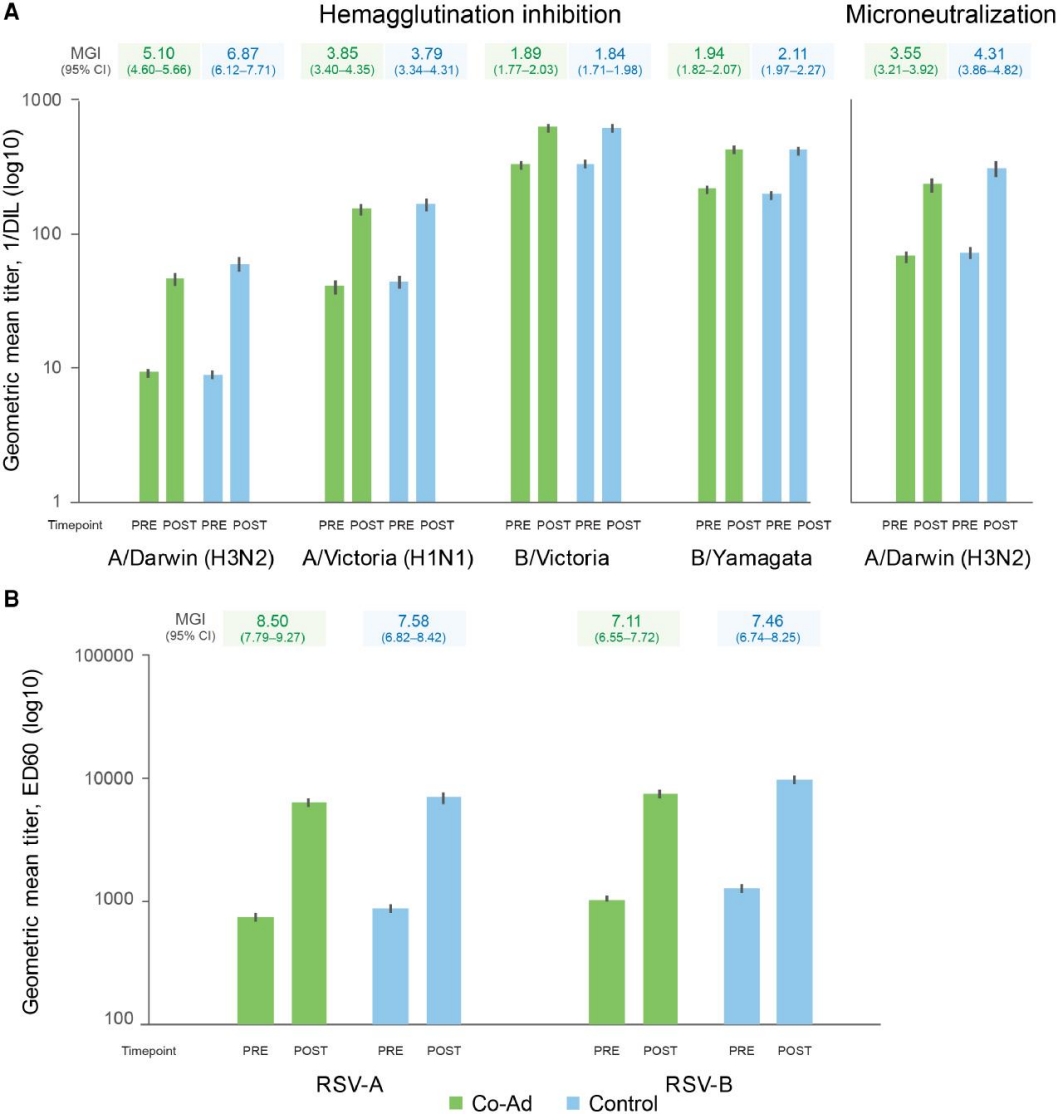
Geometric mean titers and mean geometric increases for influenza strains (*A*, per-protocol set for flu analysis) and for RSV-A and RSV-B neutralization titers (*B*, per-protocol set for RSV analysis). Co-Ad, group of participants who received RSVPreF3 OA and FLU-aQIV concomitantly on day 1; Control, group of participants who received FLU-aQIV on day 1 and RSVPreF3 OA on day 31; pre, before FLU-aQIV vaccination (day 1) for flu analysis or before RSVPreF3 OA vaccination (day 1 for Co-Ad group, day 31 for Control group) for RSV analysis; post, 1 m after FLU-aQIV vaccination (day 31) for flu analysis or 1 m after RSVPreF3 OA vaccination (day 31 for Co-Ad group, day 61 for Control group) for RSV analysis. Abbreviations: 1/DIL, 1/dilution; CI, confidence interval; ED60, estimated dilution 60; FLU-aQIV, adjuvanted inactivated seasonal quadrivalent influenza vaccine; MGI, mean geometric increase of the post- versus the pre-vaccination titers; RSVPreF3 OA, respiratory syncytial virus (RSV) prefusion F protein-based vaccine.

SCRs for HI antibodies 1 month after co-administration were similar to those after sequential administration for all influenza strains (based on the reference criterion that ULs of 95% CIs for group differences were ≤10%) except for A/Darwin (H3N2) (UL of 95% CI was 16.90%) (Table 2). SPRs were similar in both groups (63.6%–100% in the Co-Ad group and 71.0%–100% in Controls at 1 month post-vaccination). HI titers increased from pre- to post-FLU-aQIV vaccination for each influenza strain, with MGIs of 1.89–5.10 in the Co-Ad group and 1.84–6.87 in Controls (Figure 4, Supplementary Table 4). Similar increases in RSV neutralization titers were observed from pre- to post-RSVPreF3 OA vaccination in both groups, with MGIs of 8.50 (RSV-A) and 7.11 (RSV-B) in the Co-Ad group and 7.58 (RSV-A) and 7.46 (RSV-B) in Controls (Figure 4, Supplementary Table 4).

**Table 2.** Seroconversion and Seroprotection Rates for Influenza Strains (Per-protocol Set for Flu Analysis)

Influenza Strain	Timepoint	Co-Ad			Control			Difference^a^ % (95% CI)
		N	n	% (95% CI)	N	n	% (95% CI)	
Hemagglutination assay								
Seroconversion rates								
A/Darwin (H3N2)	Post	429	222	51.7 (46.9, 56.6)	400	248	62.0 (57.0, 66.8)	10.25 (3.50, 16.90)
A/Victoria (H1N1)	Post	420	185	44.0 (39.2, 48.9)	396	181	45.7 (40.7, 50.8)	1.66 (−5.16, 8.47)
B/Victoria	Post	429	74	17.2 (13.8, 21.2)	400	70	17.5 (13.9, 21.6)	0.25 (−4.92, 5.46)
B/Yamagata	Post	428	79	18.5 (14.9, 22.5)	400	77	19.3 (15.5, 23.5)	0.79 (−4.54, 6.17)
Seroprotection rates								
A/Darwin (H3N2)	Pre	469	49	10.4 (7.8, 13.6)	449	48	10.7 (8.0, 13.9)	NA
	Post	442	281	63.6 (58.9, 68.1)	400	284	71.0 (66.3, 75.4)	NA
A/Victoria (H1N1)	Pre	460	276	60.0 (55.4, 64.5)	446	287	64.3 (59.7, 68.8)	NA
	Post	440	405	92.0 (89.1, 94.4)	399	380	95.2 (92.7, 97.1)	NA
B/Victoria	Pre	469	467	99.6 (98.5, 99.9)	449	449	100 (99.2, 100)	NA
	Post	442	442	100 (99.2, 100)	400	400	100 (99.1, 100)	NA
B/Yamagata	Pre	468	467	99.8 (98.8, 100)	449	444	98.9 (97.4, 99.6)	NA
	Post	442	442	100 (99.2, 100)	400	400	100 (99.1, 100)	NA
Microneutralization assay (post-hoc)								
Seroconversion rates								
A/Darwin (H3N2)	Post	428	187	43.7 (38.9, 48.5)	398	193	48.5 (43.5, 53.5)	4.80 (−2.00, 11.57)
Seroprotection rates								
A/Darwin (H3N2)	Pre	469	359	76.5 (72.4, 80.3)	448	352	78.6 (74.5, 82.3)	NA
	Post	441	425	96.4 (94.2, 97.9)	399	388	97.2 (95.1, 98.6)	NA

Seroconversion rate was defined as the percentage of participants with a hemagglutination inhibition or microneutralization pre-vaccination titer <1:10 and a post-vaccination titer ≥1:40, or a pre-vaccination titer ≥1:10 and a ≥ 4-fold increase in post-vaccination titer. Seroprotection rate was defined as the percentage of participants with a hemagglutination inhibition or microneutralization titer ≥1:40.

Abbreviations: CI, confidence interval; Co-Ad, group of participants who received RSVPreF3 OA and FLU-aQIV concomitantly on day 1; Control, group of participants who received FLU-aQIV on day 1 and RSVPreF3 OA on day 31; FLU-aQIV, adjuvanted inactivated seasonal quadrivalent influenza vaccine; N, for seroconversion rates: number of participants with both pre- (day 1) and post-FLU-aQIV vaccination (day 31) results available; for seroprotection rates: number of participants with results available at the indicated timepoint; NA, not assessed; n/%, number/percentage of participants who seroconverted or were seroprotected; pre, before FLU-aQIV vaccination (day 1); post, 1 month after FLU-aQIV vaccination (day 31); RSVPreF3 OA, respiratory syncytial virus (RSV) prefusion F protein-based vaccine.

^a^Reference criterion for non-inferiority of co-administration versus sequential administration (descriptive secondary endpoint for hemagglutination inhibition, post-hoc exploratory analysis for microneutralization): upper limit of the 2-sided 95% CI for the difference in seroconversion rates (Control minus Co-Ad) was ≤10% at 1 m post-FLU-aQIV vaccination (ie, day 31 for both groups).

CBER criteria were met in terms of SPRs (LLs of 95% CIs ≥60%) for all strains in both groups, except for A/Darwin (H3N2) in the Co-Ad group (Table 2). In terms of SCRs, CBER criteria were met (LLs of 95% CIs ≥30%) for both influenza A strains but not for the B strains in the 2 groups. CHMP criteria for SPRs were met for all strains in both groups (point estimates >60%, Table 2). For SCRs, CHMP criteria were met for the A strains in both groups (point estimates >30%). For MGIs, criteria were met for the A strains in both groups and for B/Yamagata in the Control group only (MGI >2.0) (Figure 4, Supplementary Table 4).

To further characterize the immune response to A/Darwin (H3N2), we used an MN assay (post-hoc testing). The adjusted MN GMT ratio for A/Darwin (H3N2) between the Control and Co-Ad groups 1 month post-vaccination was 1.23 (95% CI: 1.06–1.42, ie, UL ≤1.50), suggesting similar post-vaccination MN titers for A/Darwin (H3N2) in both groups (Figure 3*B*). In addition, 1 month post-vaccination, MN SCRs were 43.7% (Co-Ad) and 48.5% (Control), MN SPRs were 96.4% (Co-Ad) and 97.2% (Control), and MGIs were 3.55 (Co-Ad) and 4.31 (Control) (Table 2, Figure 4, Supplementary Table 4).

### Reactogenicity and Safety

**Figure 5.**
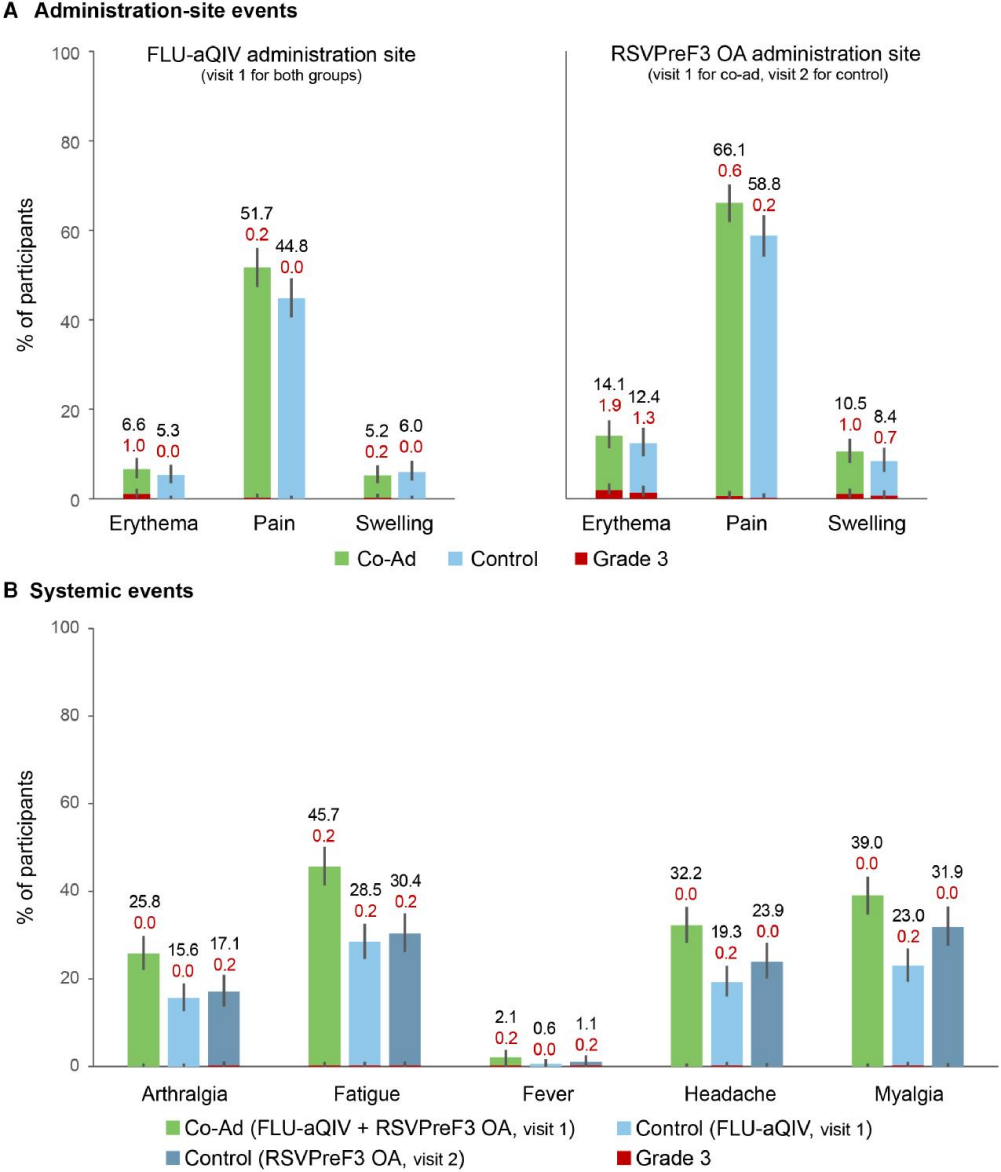
Solicited administration-site *A*, and systemic *B*, adverse events with onset within 7 d after FLU-aQIV and RSVPreF3 OA co-administration or sequential administration (exposed set). Confidence intervals are depicted as error bars. Fever was defined as a temperature ≥38.0 °C. Grade 3 adverse events were defined as administration-site erythema or swelling with a diameter >100 mm, fever with a temperature >39.0 °C, administration-site pain, arthralgia, fatigue, headache, and myalgia that prevented normal activity. Co-Ad, group of participants who received RSVPreF3 OA and FLU-aQIV concomitantly on day 1 (visit 1); Control, group of participants who received FLU-aQIV on day 1 (visit 1) and RSVPreF3 OA on day 31 (visit 2). Abbreviations: FLU-aQIV, adjuvanted inactivated seasonal quadrivalent influenza vaccine; MGI, mean geometric increase; RSVPreF3 OA, respiratory syncytial virus (RSV) prefusion F protein-based vaccine.

The most frequently reported solicited administration-site AE was pain in the 2 groups, both at the FLU-aQIV site (Co-Ad: 51.7%; Control: 44.8%) and RSVPreF3 OA site (Co-Ad: 66.1%; Control: 58.8%) (Figure 5). The most common solicited systemic AEs were fatigue and myalgia in both groups; fatigue was reported in 45.7% Co-Ad versus 28.5% (post-FLU-aQIV) and 30.4% (post-RSVPreF3 OA) Control participants and myalgia in 39.0% Co-Ad versus 23.0% (post-FLU-aQIV) and 31.9% (post-RSVPreF3 OA) Control participants (Figure 5). The median duration of solicited AEs was ≤2 days in both groups. Grade 3 solicited AEs were uncommon; the most frequently reported grade 3 solicited AE was administration-site erythema at the RSVPreF3 OA site (Co-Ad: 1.9%; Control: 1.3%), whereas grade 3 solicited systemic AEs were reported for no more than 0.2% of participants for any of the AEs in either group after any dose (Figure 5).

Overall, 13.6% Co-Ad and 24.5% Control participants reported ≥1 unsolicited AE within 30 days after any dose (Table 3), nasopharyngitis (2.5% and 2.9%), and coronavirus disease 2019 (COVID-19) (2.1% and 2.7%) being most common. Unsolicited AEs considered related to vaccination by the investigator were reported by 2.1% Co-Ad and 2.7% Control participants; myalgia was the most common in the Co-Ad group (0.4%) and fatigue, headache, and influenza-like illness in the Control group (0.4% each). Between vaccination and study end (∼6 months post-last dose), 4.0% Co-Ad and 6.9% Control participants reported SAEs, and no Co-Ad and 0.6% Control participants reported pIMDs (Table 3). One SAE in the Control group (0.2%, giant cell arteritis, also reported as pIMD) was considered by the investigator as vaccination-related; it started 10 days after the participant received FLU-aQIV and was resolving by study end; this participant did not receive RSVPreF3 OA. The other pIMDs—pericarditis and worsening of pre-existing psoriasis—were not considered vaccination-related. Fatal SAEs were reported for 6 participants, all in the Control group (1.1%) (Supplementary Table 5). None of the fatal SAEs were considered by the investigator as vaccination-related.

**Table 3.** Summary of Adverse Events (Exposed Set)

Adverse Event	Co-Ad N = 523		Control N = 522	
	n	% (95% CI)	n	% (95% CI)
Unsolicited AEs within 30 d				
Any	71	13.6 (10.8, 16.8)	128	24.5 (20.9, 28.4)
Grade 3	1	0.2 (0.0, 1.1)	6	1.1 (0.4, 2.5)
Related	11	2.1 (1.1, 3.7)	14	2.7 (1.5, 4.5)
Grade 3 related	0	0.0 (0.0, 0.7)	1	0.2 (0.0, 1.1)
Medically attended	35	6.7 (4.7, 9.2)	66	12.6 (9.9, 15.8)
SAEs and pIMDs up to study end^a^				
Any SAE	21	4.0 (2.5, 6.1)	36	6.9 (4.9, 9.4)
Related SAE	0	0.0 (0.0, 0.7)	1	0.2 (0.0, 1.1)
Fatal SAE	0	0.0 (0.0, 0.7)	6	1.1 (0.4, 2.5)
Any pIMD	0	0.0 (0.0, 0.7)	3	0.6 (0.1, 1.7)
Related pIMD	0	0.0 (0.0, 0.7)	1	0.2 (0.0, 1.1)

A grade 3 adverse event (AE) is an AE that prevents normal activity.

Abbreviations: CI, confidence interval; Co-Ad, group of participants who received RSVPreF3 OA and FLU-aQIV concomitantly on day 1; Control, group of participants who received FLU-aQIV on day 1 and RSVPreF3 OA on day 31; FLU-aQIV, adjuvanted inactivated seasonal quadrivalent influenza vaccine; N, number of participants in the exposed set; n/%, number/percentage of participants in the indicated category; pIMD, potential immune-mediated disease; RSVPreF3 OA, respiratory syncytial virus (RSV) prefusion F protein-based vaccine; SAE, serious adverse event.

^a^Approximately 6 m after the last dose.

## DISCUSSION

This study showed that co-administration of FLU-aQIV and RSVPreF3 OA was non-inferior to their sequential administration in terms of HI titers for the A/Victoria (H1N1), B/Victoria, and B/Yamagata influenza strains and in terms of RSV-A neutralization titers. Non-inferiority for A/Darwin (H3N2) was marginally missed. As the primary objectives were analyzed sequentially, the demonstration of non-inferiority for RSV-B became a descriptive endpoint; RSV-B neutralization titers were comparable after co-administration versus sequential administration. In addition, SPRs and MGIs in HI titers for each influenza strain and MGIs in RSV-A and RSV-B neutralization titers were comparable after co-administration versus sequential administration.

The non-inferiority criterion for A/Darwin (H3N2) was not met, which could be due to the low HI GMTs observed for this strain in both groups compared to the 3 other influenza strains both pre- and post-vaccination. A small difference between groups in these low GMTs could have a substantial impact when calculating GMT ratios. The low HI titers observed for A/Darwin (H3N2) may be linked to the rapid mutation rate of A/H3N2 that can result in antigenic drift, blunting the immune response. Alternatively, the low HI titers could be related to the A/H3N2 HI assay [31]. The HI assay—which detects hemagglutinin-binding antibodies that prevent influenza-mediated agglutination of red blood cells—is a standard assay used in clinical trials to quantify the immune response to influenza vaccination [29, 32]. However, several challenges have arisen with using the HI assay for the characterization of A/H3N2 strains [31, 33]. These are mainly due to the extensive genetic and antigenic evolutionary changes in A/H3N2 viruses, resulting in hemagglutinin variants with different preferences and affinity in red blood cell receptor binding, and in the ability of neuraminidase rather than hemagglutinin to agglutinate red blood cells [31]. Therefore, we further assessed the immune response to A/H3N2 by using an MN assay (post-hoc), which can overcome the aforementioned challenges with the HI assay [31]. Overall, the GMTs and SPRs for A/Darwin (H3N2) observed with the MN assay were higher than those observed with the HI assay both pre- and 1 month post-FLU-aQIV vaccination, whereas SCRs were similar for the 2 assays. The adjusted MN GMT ratio (Control/Co-Ad) 1 month post-vaccination was 1.23, with a 95% CI UL ≤1.50. Based on the MN assay, GMTs, MGIs, SPRs, and SCRs for A/Darwin (H3N2) were similar in the Co-Ad and Control groups, supporting an adequate immune response to the A/Darwin (H3N2) vaccine strain after co-administration.

Co-administration of FLU-aQIV and RSVPreF3 OA was well tolerated with an acceptable safety profile. Solicited systemic AEs were reported more frequently after co-administration than after each vaccine separately. However, this was not considered clinically relevant as no increase in severity or duration of these events was observed in the Co-Ad group. The rates of solicited administration-site AEs, unsolicited AEs, SAEs, and pIMDs were generally balanced between groups, and no clustering of AEs was noted. There were no reports of Guillain-Barré syndrome, acute disseminated encephalomyelitis, or other demyelinating disorders.

Our findings on the immunogenicity and safety of FLU-aQIV and RSVPreF3 OA co-administration add to the results of 2 previous studies that showed that RSVPreF3 OA could be safely co-administered with 2 non-adjuvanted influenza vaccines—a standard-dose and a high-dose seasonal quadrivalent influenza vaccine—without interfering with the immune responses to the vaccines [23, 24]. A recent prospective study showed that co-administration of RSVPreF3 OA with a standard-dose seasonal quadrivalent influenza vaccine was well tolerated in patients with high risk of heart failure, with no safety concerns [34]. As adjuvanted influenza vaccines are recommended in older adults to overcome immunosenescence [16], our results are of particular relevance to clinical practice in older adults.

A limitation of our study is its open-label design, which might have led to detection bias in the reactogenicity/safety assessment. Another limitation is the exclusion of persons with immunocompromising conditions, who are also at increased risk of severe RSV and influenza disease [2, 6, 10]. Finally, a relatively large proportion of participants were excluded from the per-protocol sets, especially in the Control group (>20%). Nevertheless, baseline characteristics remained balanced between groups in the per-protocol sets, and immunogenicity analyses in the exposed set showed similar results as in the per-protocol set, indicating that the eliminations due to protocol deviations did not impact our conclusions.

In summary, this study showed that the adjuvanted FLU-aQIV and RSVPreF3 OA vaccines can be co-administered in adults aged ≥65 years without clinically relevant interference with the immune responses to either vaccine and with a clinically acceptable safety and reactogenicity profile. Given the seasonal overlap of these 2 infections, the ability to give both vaccines during a single doctor's visit may improve convenience and increase uptake of both vaccines, ultimately reducing the high burden of both influenza and RSV disease among older adults.

## Supplementary Data

Supplementary materials are available at *Clinical Infectious Diseases* online. Consisting of data provided by the authors to benefit the reader, the posted materials are not copyedited and are the sole responsibility of the authors, so questions or comments should be addressed to the corresponding author.

## Supplementary Material

ciae365_Supplementary_Data

## Notes

***Author Contributions*.** N. M., M.-P. D., and S. K. were involved in the conception or design of the study/project. R. C., S. D., J. L., P. L., S. N. M., H. M. M., J. -F. N., M. P. V., M. R., M. H. R.-R., and I. S.-M, participated in the acquisition or generation of the data. S. G., M.-P. D., A. J., H. A. H., and S. K. performed the data analysis. N. D., S. G., M.-P. D., H. A. H., S. K., and N. M. were involved in the interpretation of the data.

***Acknowledgments.*** The authors are grateful to all participants, study team members, investigators, and study site staff for their contributions to the study, to Till Schoofs for his contribution to the study design, and Mohamed Oujaa for his contribution to the statistical analysis plan and analyses. They also thank CSL Seqirus for the productive collaboration on this study and Akkodis Belgium (c/o GSK) for medical writing (by Natalie Denef), design, and publication coordination support.

***Data sharing statement*.** Anonymized data can be requested from www.clinicalstudydatarequest.com.

***Trademarks*.** *Arexvy* and AS01_E_ are trademarks licensed to or owned by GSK. *Fluad Tetra, FLUAD QUADRIVALENT*, and *Fluad Quad* are registered trademarks of Seqirus.

***Financial support.*** This work was supported by GlaxoSmithKline Biologicals SA who funded this trial, was involved in all stages of trial conduct, and took charge of all costs associated with the development and publication of this manuscript.
