# Supplementary material for: Safety and Immunogenicity of Respiratory Syncytial Virus Prefusion F Protein Vaccine when Co-administered with Adjuvanted Seasonal Quadrivalent Influenza Vaccine in Older Adults: A Phase 3 Randomized Trial
Source: Clin Infect Dis. 2024 Aug 5;79(4):1088–98. doi: 10.1093/cid/ciae365 (PMC11478588; doi:10.1093/cid/ciae365)
Supplement: ciae365_Supplementary_Data [file ciae365_supplementary_data.docx]

**Supplementary material**

**Supplementary methods**

***Inclusion criteria***

All participants were to satisfy all the following criteria at study entry:

- Participants who, in the opinion of the investigator, could and would comply with the requirements of the protocol (e.g., completion of the electronic diary cards [eDiaries], return for follow-up visits, ability to access and use a phone or other electronic communications).
  - Note: In case of physical incapacity that would preclude the self-completion of the eDiaries, either site staff could assist the participant (for activities performed during site visits) or the participants could assign a caregiver to assist them with this activity (for activities performed at home). However, at no time could the site staff or caregiver evaluate the participant’s health status while completing diaries or make decisions on behalf of the participant.
- A man or woman ≥65 years of age at the time of the first study intervention administration.
- Participants living in the general community or in an assisted-living facility that provided minimal assistance, such that the participant was primarily responsible for self-care and activities of daily living.
- Written or witnessed informed consent obtained from the participant prior to performance of any study-specific procedure.
- Participants who were medically stable in the opinion of the investigator at the time of first study intervention administration. Participants with chronic stable medical conditions with or without specific treatment, such as diabetes, hypertension, or cardiac disease, were allowed to participate in this study if considered by the investigator as medically stable*.*

***Exclusion criteria***

A person could not be included in the study if any of the following exclusion criteria applied:

- Any confirmed or suspected immunosuppressive or immunodeficient condition resulting from disease (e.g., current malignancy, human immunodeficiency virus) or immunosuppressive/cytotoxic therapy (e.g., medication used during cancer chemotherapy, organ transplantation, or to treat autoimmune disorders), based on medical history and physical examination (no laboratory testing required).
- History of any reaction or hypersensitivity (e.g., anaphylaxis) likely to be exacerbated by any component of the study interventions, in particular any history of severe allergic reaction to egg protein or to a previous influenza vaccine.
- Hypersensitivity to latex.
- Guillain-Barré syndrome that occurred within 6 weeks of receipt of prior influenza vaccine.
- Serious or unstable chronic illness.
- Any history of dementia or any medical condition that moderately or severely impairs cognition.
  - Note: If deemed necessary for clinical evaluation, the investigator could use tools such as Mini-Mental State Examination, Mini-Cog or Montreal Cognitive Assessment to determine cognition levels of the participant.
- Recurrent or uncontrolled neurological disorders or seizures. Participants with medically controlled active or chronic neurological diseases could be enrolled in the study as per investigator assessment, provided that their condition would allow them to comply with the requirements of the protocol (e.g., completion of the eDiaries, attend regular phone calls/study site visits).
- Significant underlying illness that in the opinion of the investigator would be expected to prevent completion of the study (e.g., life-threatening disease likely to limit survival up to study end).
- Any medical condition that in the judgment of the investigator would make intramuscular injection unsafe.
- Use of any investigational or non-registered product (drug, vaccine or medical device) other than the study interventions during the period beginning 30 days before the first dose of study interventions, or planned use during the study period.
- Administration of an influenza vaccine during the 6 months preceding the study influenza vaccine administration.
- Planned or actual administration of a vaccine not foreseen by the study protocol in the period starting 30 days before the first study intervention administration and ending 30 days after the last study intervention administration. In the case of COVID-19 vaccines, this time window could be decreased to 14 days before and after each study intervention administration provided this COVID-19 vaccine use was in line with local governmental recommendations.
  - Note: In case an emergency mass vaccination for an unforeseen public health threat (e.g., a pandemic) was recommended and/or organized by the public health authorities outside the routine immunization program, the time period described above could be reduced if necessary for that vaccine, provided it was used according to the local governmental recommendations and that the Sponsor was notified accordingly.
- Previous vaccination with a respiratory syncytial virus (RSV) vaccine.
- Administration of long-acting immune-modifying drugs or planned administration at any time during the study period (e.g., infliximab).
- Administration of immunoglobulins and/or any blood products or plasma derivatives during the period starting 90 days before the administration of the first dose of the study interventions or planned administration during the study period.
- Chronic administration (defined as more than 14 consecutive days in total) of immunosuppressants or other immune-modifying drugs during the period starting 90 days prior to the first study intervention dose or planned administration during the study period. For corticosteroids, this meant prednisone ≥20 mg/day, or equivalent. Inhaled and topical steroids were allowed.
- Concurrently participating in another clinical study, at any time during the study period, in which the participant had been or would be exposed to an investigational or a non-investigational vaccine/product (drug or invasive medical device).
  - Note: European Economic Community directive 93/42/EEC defines an invasive medical device as “A device which, in whole or in part, penetrates inside the body, either through a body orifice or through the surface of the body”.
- History of chronic alcohol consumption and/or drug abuse as deemed by the investigator to render the potential participant unable/unlikely to provide accurate safety reports or comply with study procedures.
- Bedridden participants.
- Planned move during the study conduct that would prohibit participation until study end.
- Participation of any study personnel or their immediate dependents, family, or household members.

***Enrollment rules***

Participants were enrolled in three age categories, with a distribution reflecting the age distribution in the general population and a balance between men and women. The aim was, therefore, to enroll:

- ~25% of participants 65–69 years old, ~40% of participants 70–79 years old, and ~10% of participants ≥80 years old; and the remaining 25% distributed freely across the three age categories.
- ~40% of participants from each sex and the remaining 20% distributed freely between the two sexes.

***Vaccine composition and randomization***

Each 0.5-ml dose of reconstituted RSV prefusion F protein-based vaccine (RSVPreF3 OA) contained 120 μg of RSVPreF3 antigen and AS01_E_, an adjuvant system containing 25 μg of 3-O-desacyl-4′-monophosphoryl lipid A, 25 μg of QS21 (*Quillaja saponaria* Molina, fraction 21, licensed by GSK from Antigenics LLC, a wholly owned subsidiary of Agenus Inc., a Delaware, USA corporation), and liposome.

Each 0.5-ml dose of the adjuvanted inactivated seasonal quadrivalent influenza vaccine (FLU-aQIV, marketed by Seqirus as *Fluad Tetra, FLUAD QUADRIVALENT,* and *Fluad Quad*) contained 15 µg hemagglutinin of each of the following strains: influenza A/Victoria/2570/2019 (H1N1)pdm09-like virus, influenza A/Darwin/9/2021 (H3N2)-like virus, influenza B/Austria/1359417/2021 (Victoria lineage)-like virus, influenza B/Phuket/3073/2013 (Yamagata lineage)-like virus., adjuvanted with MF59C.1 (squalene: 9.75 mg, polysorbate 80: 1.175 mg, sorbitan trioleate: 1.175 mg). The influenza vaccine strains were those recommended by the World Health Organization for the 2022/2023 Northern-Hemisphere influenza season [1].

Participants were randomized using an automated internet-based system. The randomization algorithm used a minimization procedure accounting for age (65–69, 70–79, or ≥80 years) and study center, with equal weight for these minimization factors.

***Laboratory assay cut-offs***

The lower and upper limits of quantification (LLOQs and ULOQs) for the different assays were:

| Assay | LLOQ | ULOQ |
| --- | --- | --- |
| **Influenza hemagglutination inhibition assay** |  |  |
| A/Darwin/6/2021 H3N2 | 1:10 | 1:2560 |
| A/Victoria/2570/2019 H1N1 | 1:10 | 1:3620 |
| B/Austria/1359417/2021 Victoria | 1:10 | 1:1810 |
| B/Phuket/3073/2013 Yamagata | 1:10 | 1:3620 |
| **RSV neutralization assay** |  |  |
| RSV-A | 18 ED60 | 123,535 ED60 |
| RSV-B | 30 ED60 | 138,336 ED60 |
| **Influenza microneutralization assay** |  |  |
| A/Darwin/6/2021 H3N2 | 1:10 | 1:10,240 |

ED60, estimated dilution 60.

***Statistical analyses***

Target enrollment was 1028 participants (514 in each group). Assuming that 10% of participants would be non-evaluable for the primary objectives, this would give 462 evaluable participants per group. Each primary objective was evaluated with a nominal type I error of 2.5% and a non-inferiority margin of 1.5. Considering a potential slight interference of 1.05 in true geometric mean titers in both groups with a common population standard error of 0.45 for the RSV-A and RSV-B neutralization titers and 0.6 for each of the FLU-aQIV strains in log_10_-transformed hemagglutinin inhibition titers, the study had at least 90.0% power to meet the primary objectives.

For the primary non-inferiority objectives, the 95% confidence intervals for the geometric mean titer ratios were calculated using an analysis of covariance model. The model included study group and age category at vaccination (65–69, 70–79, or ≥80 years) as fixed effects, and the pre-vaccination log_10_-transformed titer as covariate. Missing data were not replaced. Titers below the LLOQs were replaced by half the LLOQs; titers above the ULOQs were replaced by the ULOQs.

For the secondary confirmatory objective, the 95% confidence intervals for the differences in seroconversion rates were calculated based on the method of Miettinen and Nurminen [2].

**Supplementary tables**

**Supplementary Table 1. Baseline characteristics of the participants (per-protocol sets for flu and RSV analyses)**

| **Characteristic** | **Per-protocol set for flu analysis** | | **Per-protocol set for RSV analysis** | |
| --- | --- | --- | --- | --- |
|  | **Co-Ad**  **N=471** | **Control**  **N=400** | **Co-Ad**  **N=471** | **Control**  **N=374** |
| Mean age (SD), years | 72.0 (5.4) | 72.2 (5.1) | 72.0 (5.4) | 72.1 (5.2) |
| Age group, n (%) |  |  |  |  |
| 65–69 years | 185 (39.3) | 154 (38.5) | 185 (39.3) | 143 (38.2) |
| 70–79 years | 240 (51.0) | 209 (52.3) | 240 (51.0) | 200 (53.5) |
| ≥80 years | 46 (9.8) | 37 (9.3) | 46 (9.8) | 31 (8.3) |
| Sex, n (%) |  |  |  |  |
| Female | 240 (51.0) | 194 (48.5) | 240 (51.0) | 176 (47.1) |
| Male | 231 (49.0) | 206 (51.5) | 231 (49.0) | 198 (52.9) |
| Race, n (%) |  |  |  |  |
| Asian | 0 (0.0) | 1 (0.3) | 0 (0.0) | 1 (0.3) |
| Black | 0 (0.0) | 0 (0.0) | 0 (0.0) | 0 (0.0) |
| White | 471 (100) | 397 (99.3) | 471 (100) | 371 (99.2) |
| Other | 0 (0.0) | 1 (0.3) | 0 (0.0) | 1 (0.3) |
| Unknown | 0 (0.0) | 1 (0.3) | 0 (0.0) | 1 (0.3) |
| Country, n (%) |  |  |  |  |
| Belgium | 58 (12.3) | 56 (14.0) | 58 (12.3) | 55 (14.7) |
| Finland | 52 (11.0) | 44 (11.0) | 52 (11.0) | 37 (9.9) |
| France | 103 (21.9) | 98 (24.5) | 103 (21.9) | 81 (21.7) |
| Spain | 203 (43.1) | 150 (37.5) | 203 (43.1) | 156 (41.7) |
| United Kingdom | 55 (11.7) | 52 (13.0) | 55 (11.7) | 45 (12.0) |

Co-Ad, group of participants who received respiratory syncytial virus prefusion F protein-based vaccine (RSVPreF3 OA) and adjuvanted inactivated seasonal quadrivalent influenza vaccine (FLU-aQIV) concomitantly on day 1; Control, group of participants who received FLU-aQIV on day 1 and RSVPreF3 OA on day 31; N, number of participants in the per-protocol sets; SD, standard deviation; n (%), number (percentage) of participants in the indicated category.

**Supplementary Table 2. Pre-existing medical conditions (exposed set)**

| **Pre-existing condition, n (%)** | **Co-Ad**  **N=523** | **Control**  **N=522** |
| --- | --- | --- |
| SOC: Respiratory, thoracic, and mediastinal disorders | 132 (25.2) | 143 (27.4) |
| PT: Chronic obstructive pulmonary disease | 46 (8.8) | 49 (9.4) |
| PT: Asthma | 41 (7.8) | 40 (7.7) |
| SOC: Cardiac disorders | 98 (18.7) | 96 (18.4) |
| SOC: Metabolism and nutrition disorders | 254 (48.6) | 249 (47.7) |
| HLT: Diabetes mellitus (incl subtypes) | 74 (14.1) | 75 (14.4) |

Pre-existing medical condition based on the participant’s medical history obtained by interviewing the participant and/or reviewing the participant’s medical records. Classification according to Medical Dictionary for Regulatory Activities Terminology system organ class (SOC), high-level term (HLT), and preferred term (PT).

n (%), number (percentage) of participants in the indicated category; Co-Ad, group of participants who received respiratory syncytial virus prefusion F protein-based vaccine (RSVPreF3 OA) and adjuvanted inactivated seasonal quadrivalent influenza vaccine (FLU-aQIV) concomitantly on day 1; Control, group of participants who received FLU-aQIV on day 1 and RSVPreF3 OA on day 31; N, number of participants in the exposed set.

**Supplementary Table 3. Adjusted geometric mean titer ratios for influenza strains, RSV-A, and RSV-B (exposed set)**

| **Assay** | **N** | | **Adjusted GMT ratio (Control/Co-Ad)** |
| --- | --- | --- | --- |
|  | **Co-Ad** | **Control** |  |
| **Hemagglutination inhibition** |  |  |  |
| A/Darwin (H3N2) | 474 | 453 | 1.37 (1.18, 1.58) |
| A/Victoria (H1N1) | 465 | 448 | 1.06 (0.93, 1.20) |
| B/Victoria | 474 | 453 | 0.97 (0.90, 1.05) |
| B/Yamagata | 473 | 453 | 1.04 (0.96, 1.13) |
| **RSV neutralization** |  |  |  |
| RSV-A | 519 | 499 | 1.03 (0.92, 1.16) |
| RSV-B | 517 | 499 | 1.18 (1.06, 1.32) |

Co-Ad, group of participants who received respiratory syncytial virus (RSV) prefusion F protein-based vaccine (RSVPreF3 OA) and adjuvanted inactivated seasonal quadrivalent influenza vaccine (FLU-aQIV) concomitantly on day 1; Control, group of participants who received FLU-aQIV on day 1 and RSVPreF3 OA on day 31; N, number of participants with results available; GMT, geometric mean titer.

**Supplementary Table 4. Geometric mean titers and mean geometric increases for influenza strains (per-protocol set for flu analysis) and for RSV-A and RSV-B neutralization titers (per-protocol set for RSV analysis)**

| **Assay** | **Timepoint** | **Co-Ad** | | |  | | **Control** | | | |  |
| --- | --- | --- | --- | --- | --- | --- | --- | --- | --- | --- | --- |
|  |  | **N** | **GMT (95% CI)** | **MGI (95% CI)** | |  | | **N** | **GMT (95% CI)** | **MGI (95% CI)** | |
| **Hemagglutination inhibition** | | | | | | | | | | | |
| A/Darwin (H3N2) | Pre | 469 | 9.1 (8.4, 9.8) | - | |  | | 449 | 8.7 (8.1, 9.4) | - | |
|  | Post | 442 | 45.3 (40.6, 50.5) | 5.10 (4.60, 5.66) | |  | | 400 | 58.3 (51.6, 65.8) | 6.87 (6.12, 7.71) | |
| A/Victoria (H1N1) | Pre | 460 | 40.0 (35.4, 45.1) | - | |  | | 446 | 43.2 (38.4, 48.5) | - | |
|  | Post | 440 | 151.0 (136.4, 167.2) | 3.85 (3.40, 4.35) | |  | | 399 | 163.8 (147.5, 181.9) | 3.79 (3.34, 4.31) | |
| B/Victoria | Pre | 469 | 323.6 (300.4, 348.5) | - | |  | | 449 | 327.2 (303.6, 352.7) | - | |
|  | Post | 442 | 614.9 (575.5, 657.0) | 1.89 (1.77, 2.03) | |  | | 400 | 608.1 (566.8, 652.5) | 1.84 (1.71, 1.98) | |
| B/Yamagata | Pre | 468 | 213.1 (198.5, 228.7) | - | |  | | 449 | 193.2 (179.9, 207.5) | - | |
|  | Post | 442 | 423.0 (394.6, 453.5) | 1.94 (1.82, 2.07) | |  | | 400 | 417.5 (387.9, 449.4) | 2.11 (1.97, 2.27) | |
| **Microneutralization** | | | | | | | | | | | |
| A/Darwin (H3N2) | Pre | 469 | 66.6 (60.4, 73.4) | - | |  | | 448 | 71.2 (64.8, 78.3) | - | |
|  | Post | 441 | 229.9 (203.6, 259.5) | 3.55 (3.21, 3.92) | |  | | 399 | 306.1 (266.4, 351.7) | 4.31 (3.86, 4.82) | |
| **RSV neutralization** | | | | | | | | | | |  |
| RSV-A | Pre | 503 | 739.9 (688.8, 794.7) | - | |  | | 447 | 894.3 (826.1, 968.2) | - | |
|  | Post | 471 | 6271.2 (5734.8, 6857.9) | 8.50 (7.79, 9.27) | |  | | 373 | 6891.0 (6208.1, 7649.1) | 7.58 (6.82, 8.42) | |
| RSV-B | Pre | 503 | 1051.7 (974.9, 1134.4) | - | |  | | 447 | 1267.7 (1167.4, 1376.8) | - | |
|  | Post | 469 | 7438.5 (6835.5, 8094.7) | 7.11 (6.55, 7.72) | |  | | 373 | 9667.6 (8715.7, 10723.3) | 7.46 (6.74, 8.25) | |

Microneutralization was tested post-hoc. Co-Ad, group of participants who received respiratory syncytial virus (RSV) prefusion F protein-based vaccine (RSVPreF3 OA) and adjuvanted inactivated seasonal quadrivalent influenza vaccine (FLU-aQIV) concomitantly on day 1; Control, group of participants who received FLU-aQIV on day 1 and RSVPreF3 OA on day 31; N, number of participants with results available at the indicated timepoint (note: N values for MGI were the number of participants with both pre- and post-vaccination results available, which differed slightly from the indicated values); GMT, geometric mean titer, expressed in 1/dilution for the influenza assays and in estimated dilution 60 for the RSV assays; CI, confidence interval; MGI, mean geometric increase of the post- versus the pre-vaccination titers; pre, before FLU-aQIV vaccination (day 1) for flu analysis or before RSVPreF3 OA vaccination (day 1 for Co-Ad group, day 31 for Control group) for RSV analysis; post, 1 month after FLU-aQIV vaccination (day 31) for flu analysis or 1 month after RSVPreF3 OA vaccination (day 31 for Co-Ad group, day 61 for Control group) for RSV analysis.

**Supplementary Table 5. Participants with serious adverse events with a fatal outcome from vaccination until study end (exposed set)**

|  | **Co-Ad**  **N=523** | |  | **Control**  **N=522** | |
| --- | --- | --- | --- | --- | --- |
| **Primary System Organ Class**  **Preferred Term** | **n** | **% (95% CI)** |  | **n** | **% (95% CI)** |
| Any fatal serious adverse event | 0 | 0.0 (0.0, 0.7) |  | 6 | 1.1 (0.4, 2.5) |
| Respiratory, thoracic, and mediastinal disorders | 0 | 0.0 (0.0, 0.7) |  | 3 | 0.6 (0.1, 1.7) |
| Chronic obstructive pulmonary disease | 0 | 0.0 (0.0, 0.7) |  | 3 | 0.6 (0.1, 1.7) |
| Pulmonary arterial hypertension | 0 | 0.0 (0.0, 0.7) |  | 2 | 0.4 (0.0, 1.4) |
| Hypercapnia | 0 | 0.0 (0.0, 0.7) |  | 1 | 0.2 (0.0, 1.1) |
| Infections and infestations | 0 | 0.0 (0.0, 0.7) |  | 2 | 0.4 (0.0, 1.4) |
| Infective exacerbation of bronchiectasis | 0 | 0.0 (0.0, 0.7) |  | 1 | 0.2 (0.0, 1.1) |
| Pneumonia | 0 | 0.0 (0.0, 0.7) |  | 1 | 0.2 (0.0, 1.1) |
| Neoplasms benign, malignant, and unspecified (incl. cysts and polyps) | 0 | 0.0 (0.0, 0.7) |  | 2 | 0.4 (0.0, 1.4) |
| Adenocarcinoma gastric | 0 | 0.0 (0.0, 0.7) |  | 1 | 0.2 (0.0, 1.1) |
| Small intestine carcinoma | 0 | 0.0 (0.0, 0.7) |  | 1 | 0.2 (0.0, 1.1) |
| Cardiac disorders | 0 | 0.0 (0.0, 0.7) |  | 1 | 0.2 (0.0, 1.1) |
| Right ventricular failure | 0 | 0.0 (0.0, 0.7) |  | 1 | 0.2 (0.0, 1.1) |
| Gastrointestinal disorders | 0 | 0.0 (0.0, 0.7) |  | 1 | 0.2 (0.0, 1.1) |
| Upper gastrointestinal hemorrhage | 0 | 0.0 (0.0, 0.7) |  | 1 | 0.2 (0.0, 1.1) |
| Nervous system disorders | 0 | 0.0 (0.0, 0.7) |  | 1 | 0.2 (0.0, 1.1) |
| Encephalopathy | 0 | 0.0 (0.0, 0.7) |  | 1 | 0.2 (0.0, 1.1) |

Note: One participant had five serious adverse events leading to death (chronic obstructive pulmonary disease, encephalopathy, hypercapnia, infective exacerbation of bronchiectasis, and pulmonary arterial hypertension), one participant had three (chronic obstructive pulmonary disease, pneumonia, and small intestine carcinoma), and one participant had two (chronic obstructive pulmonary disease and pulmonary arterial hypertension). The others each had one serious adverse event with fatal outcome.

Co-Ad, group of participants who received respiratory syncytial virus prefusion F protein-based vaccine (RSVPreF3 OA) and adjuvanted inactivated seasonal quadrivalent influenza vaccine (FLU-aQIV) concomitantly on day 1; Control, group of participants who received FLU-aQIV on day 1 and RSVPreF3 OA on day 31; N, number of participants in the exposed set; n/%, number/percentage of participants with the event; CI, confidence interval.

**Supplementary references**

1. World Health Organization. Recommended composition of influenza virus vaccines for use in the 2022-2023 northern hemisphere influenza season. Available at: <https://www.who.int/publications/m/item/recommended-composition-of-influenza-virus-vaccines-for-use-in-the-2022-2023-northern-hemisphere-influenza-season>. Accessed 15 February 2024.

2. Miettinen O, Nurminen M. Comparative analysis of two rates. Stat Med **1985**; 4:213-226.
